# Research Letter: Limited additional serious adverse events associated with concomitant immunomodulatory treatment in people with atypical psychiatric disease

**DOI:** 10.1177/00048674241271969

**Published:** 2024-09-03

**Authors:** Parisa Fani-Molky, Jocelyn Jiang, Sabrina Naz, David Brown, Anthony Harris

**Affiliations:** 1 Sydney Medical School, The University of Sydney, Sydney, NSW, Australia; 2 Western Sydney University, Westmead, NSW, Australia; 3 Western Sydney Local Health District, Westmead, NSW, Australia; 4 Blacktown Hospital, Blacktown, NSW, Australia; 5 Douglass Hanly Moir Pathology, Macquarie Park, NSW, Australia; 6 Westmead Hospital, Westmead, NSW, Australia; 7 Research and Education Network (REN), Western Sydney Local Health District, Westmead, NSW, Australia; 8 The Westmead Institute for Medical Research, Westmead, NSW, Australia; 9 Centre for Immunology and Allergy Research, Institute for Clinical Pathology and Medical Research, NSW Health Pathology, Newcastle, NSW, Australia

## Introduction

There is increasing interest in the role of the immune/inflammatory systems in psychiatric disease (Ellul et al., 2017). This has been contributed to by the increasing recognition of autoimmune encephalitis (AIE), where psychiatric symptoms are part of the clinical presentation and may be the only symptom. While classically, diagnosis of AIE is based on the presence of specific antibodies (Bost et al., 2016), antibody-negative AIE is now well recognized (Jiang et al., 2020; Pollak et al., 2020).

Treatment-resistant psychiatric symptoms may be contributed to by ongoing inflammatory-immune mechanisms (Pollak et al., 2020). Support for an inflammatory mechanism also comes from the observation that commonly used psychotropics have exhibited anti-inflammatory effects in humans (Patel et al., 2023), and clozapine has clear evidence of immunomodulation in animal models of neuroinflammatory disease (Al Abadey et al., 2022; Robichon et al., 2021). While augmentation of therapy in psychiatric disease has been examined in the context of anti-inflammatory therapy (Jeppesen et al., 2020), treatments that directly target immune cellular systems are less studied and the frequency of serious adverse events (SAEs) is of concern. Notably, psychiatric patients often experience adverse events (AEs) with psychotropics, driving concerns that adding immunotherapy such as glucocorticoids can further exacerbate their vulnerabilities. In addition, there is caution when combining multiple treatment modalities with weak supporting evidence. Therefore, it is imperative to frequently audit patients to ensure the risk–benefit ratio of individualized therapeutic trials is balanced towards effective treatment.

To determine the safety of immunosuppressive treatments in this treatment group, we conducted a retrospective review of the electronic medical records (eMR) of patients referred to the Immunology Service at a tertiary hospital for assessment of the possibility of an immune contribution to their psychiatric diseases.

## Methods

Adult patients over 18 years old with atypical and/or treatment-resistant psychiatric disease undergoing immunology team review at a large tertiary hospital were included in retrospective review of their eMR. Referrals were made from psychiatrists for further immunological testing due to clinical suspicion and/or positive serum autoantibodies. Data were collected from the patient’s initial visit date with an immunologist from April 2013 to November 30, 2023. It was then entered into a University of Sydney Research Electronic Data Capture (REDCap) database. As a retrospective review of eMR, informed consent from individual patients was not obtained. This study was approved as a Quality Assurance project by the Western Sydney Local Health District Human Research Ethics Committee (2023/ETH02171).

Results of brain imaging, electroencephalogram (EEG) and cerebrospinal fluid were obtained from medical records. The clinical global impression severity (CGI-S) rating scale was used to measure symptom severity at time of first contact with the immunology team (baseline) and final visit. The CGI-S score was determined independently by P.F.-M., D.B. and A.H., and a consensus was reached. AEs were systematically categorized using the Common Terminology Criteria for Adverse Events v5.0 (US Department of Health and Human Services, 2017). SAEs were classified as AEs that required hospitalization. The Australian Medicines Handbook and expert advice were consulted to determine established AEs for each specific therapy.

Descriptive statistics were employed to summarize demographics, diagnostic information and treatment modalities. Frequencies and percentages were used for categorical variables and median and interquartile range for continuous variables. A linear mixed-effects model for CGI-S score with a general positive definite covariance structure was used to investigate the association between the change in CGI-S score and immunotherapy status. Patient identifier was considered as a random effect and time (baseline/final) as both a fixed and random effect. The interaction between the fixed effects of time and immunotherapy status was used to test for evidence that the change in CGI-S score differed by treatment. The estimated marginal means and their 95% confidence intervals (CIs) were used to summarize the CGI-S scores observed at each time period by treatment. Chi-square tests were used to test for association between the worst grade of AEs experienced by a patient and possible causative medication.

## Results

**Figure 1.**
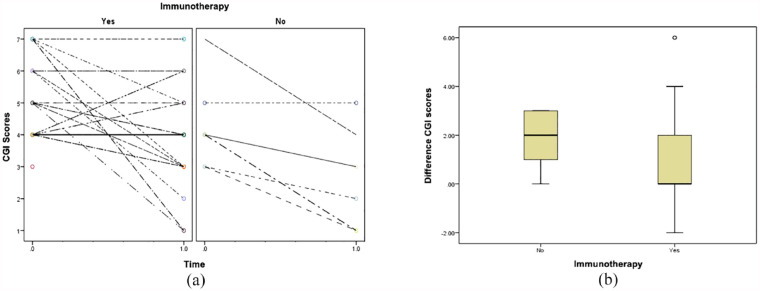
Clinical Global Impression Severity (CGI-S) scores at baseline compared to the final visit, stratified by immunotherapy status (*n* = 36). The analysis includes *n* = 29 patients receiving immunotherapy and *n* = 7 not receiving immunotherapy, excluding cases with unknown final CGI-S scores (*n* = 6). (a) Individual patient profile plots of CGI-S score by time, with zero indicating baseline and 1.0 representing the final visit. Lines connect individual trajectories and (b) boxplot illustrating the distribution of the within patient change in CGI score (baseline–final) by immunotherapy status (yes/no). A linear mixed-effects model for CGI-S score with a general positive definite covariance structure was used to investigate the association between the change in CGI-S score and immunotherapy status. Patient identifier was considered as a random effect and time (baseline/final) as both a fixed and as a random effect. The interaction between the fixed effects of time and immunotherapy status was used to test for evidence that the change in CGI-S score differed by treatment. This interaction term was not significant (*p* = 0.256). Estimated marginal means and their 95% confidence intervals were used to summarize the CGI-S scores observed at each time by treatment.

Full details of the clinical characteristics, treatment modalities and AEs profiles of study patients are outlined in Table 1. Individual patient profile plots of CGI-S scores at baseline and final visit by immunotherapy status are shown in Figure 1(a). Boxplots illustrating the change in CGI-S scores (baseline–final visit) by immunotherapy status are shown in Figure 1(b). The estimated marginal means of the CGI-S score in patients receiving immunotherapy were 5.00 (95% CI = 4.52–5.48) at baseline and 4.09 (95% CI = 3.57–4.62) at final visit. The estimated marginal means in patients who did not receive immunotherapy were 4.29 (95% CI = 3.22–5.35) at baseline and 2.43 (95% CI = 1.37–3.49) at final visit. The linear mixed-effects model did not detect a significant association between the change in CGI-S score and immunotherapy status (interaction *p* = 0.256). The mean changes observed in the no immunotherapy (*n* = 7) and immunotherapy (*n* = 29) groups were 1.9 (95% CI = 0.7–3.0) and 1.0 (95% CI = 0.2–1.8), respectively, *p* < 0.02 in both groups.

**Table 1.** Clinical characteristics, treatment modalities and adverse events profile in study patients (*n* = 42).

Variable	Median (IQR)	Range
Age	25.5 (22.3–28.0)	18–66
Time to initial immunology visit in years	3.5 (1.0–7.8)	0–18
Variable	*n*	(%)
Sex		
Male	8	19.0
Female	34	81.0
Predominant psychiatric presentation		
Psychotic	15	35.7
Mood	17	40.5
Behavioural	1	2.4
Anxiety	2	4.8
Cognitive	6	14.3
Other	1	2.4
Psychiatric diagnosis	42	100.0
>1 psychiatric diagnosis	33	78.6
Autoimmune diagnosis	23	54.8
>1 autoimmune diagnosis	9	21.4
Childhood psychiatric diagnosis	23	54.8
Family history		
Autoimmune disease	10	23.8
Psychiatric disease	11	26.2
Both autoimmune and psychiatric disease	10	23.8
No family history of autoimmune and psychiatric disease	6	14.3
Exact family history not known	5	11.9
Brain imaging		
Brain imaging normal	15	35.7
Brain imaging abnormal	25	59.5
*MRI abnormality only*	*4*	*9.5*
*SPECT abnormality only*	*18*	*42.9*
*Both MRI and SPECT abnormality*	*3*	*7.1*
Imaging not done	1	2.4
Imaging result not available	1	2.4
EEG		
EEG normal	18	42.9
EEG abnormal	9	21.4
EEG not done	14	33.3
EEG result not available	1	2.4
Number of serum autoantibodies		
Absent	6	14.3
1	15	41.7
2	12	33.3
3	5	13.9
4	1	2.8
5+	3	8.3
Variable	*n*	(%)
CSF		
Normal	14	33.3
Abnormal	27	64.3
Refused	1	2.4
Psychotropic classes trialled^a^		
Antidepressants	32	76.2
Antipsychotics	36	85.7
Mood stabilizers	18	42.9
Benzodiazepines	16	38.1
Stimulants	7	16.7
Lithium	18	42.9
Clozapine	9	21.4
Other	3	7.1
Number of psychotropic classes trialled per patient		
1	9	21.4
2	7	16.7
3	7	16.7
4	10	23.8
5	2	4.8
6	4	9.5
7	3	7.1
Immunotherapy treatment trialled^a^		
Treated with immunotherapy	34	81.0
*Glucocorticoids*	20	47.6
*Minocycline*	19	45.2
*Mycophenolate*	16	38.1
*IVIg*	13	31.0
*Methotrexate*	12	28.6
*Hydroxychloroquine*	12	28.6
*Sirolimus*	8	19.0
*Rituximab*	7	16.7
*Cyclophosphamide*	1	2.4
*Plex*	2	4.8
*Azathioprine*	2	4.8
*Other*	1	2.4
Not indicated or refused	8	19.0
Number of immunotherapy medications trialled per patient		
0	8	19.0
1	7	16.7
2	9	21.4
3	3	7.1
4	7	16.7
5	1	2.4
6	4	9.5
7	3	7.1
AEs categorized by causative agent^b^	117	100.0
Immunotherapy only	58	49.6
*Infections and infestations*	22	18.8
Variable	*n*	(%)
*Gastrointestinal disorders*	14	12.0
*Endocrine disorders*	5	4.3
*Other*	17	14.5
Psychotropics	40	34.2
*Nervous system disorders*	13	11.1
*Investigations*	11	9.4
*Gastrointestinal disorders*	7	6.0
*Other*	9	7.7
Both immunotherapy and psychotropics	7	6.0
*Investigations*	5	4.3
*Other*	2	1.7
None	12	10.2
*Gastrointestinal disorders*	3	2.6
*Infections and infestations*	3	2.6
*Other*	6	5.0
Worst grade of AE by medications classes for patients who experienced at least one AE	*p* = 0.661	
Grade 1	8	
*Immunotherapy only*	3	37.5
*Psychotropic only*	3	37.5
*Both*	2	25.0
*None*	0	0.0
Grade 2	13	
*Immunotherapy only*	5	38.5
*Psychotropic only*	4	30.8
*Both*	1	7.7
*None*	3	23.1
Grade 3	8	
*Immunotherapy only*	4	50.0
*Psychotropic only*	2	25.0
*Both*	0	0.0
*None*	2	25.0
Grade 4	3	
*Immunotherapy only*	2	66.7
*Psychotropic only*	0	0.0
*Both*	1	33.3
*None*	0	0.0
Ongoing concerns^c^		
Mental health^d^	15	35.7
Chronic fatigue	7	16.7
Chronic pain	6	14.3
Cognition	6	14.3
Postural orthostatic tachycardia syndrome	2	4.8
No concerns	5	11.9
Not known	8	19.0
Lost to follow-up	15	37.5

AE: adverse event, CSF: cerebrospinal fluid; EEG: electroencephalogram; IQR: interquartile range; MRI: magnetic resonance imaging; SPECT: single-photon emission computed tomography.

a Numbers will not total to 100%, as some patients have received multiple medications at once.

b Categories are determined using the Common Terminology Criteria for Adverse Events v5.0 (US Department of Health and Human Services, 2017).

c Numbers will not total to 100%, as some patients may have multiple concerns.

d Mental health concerns range from a variety of psychiatric symptoms such as ongoing depression, anxiety and psychosis.

AEs occurred over 129.7 cumulative treatment years across all patients, with a total of 117 reported AEs. Of the 42 patients, 10 did not report any AEs, while four patients contributed to 47 (40%) of the total AEs. The majority of reported AEs were classified as grade 1 (35.0%) or grade 2 (45.3%), while 13.7% of all AEs were categorized as grade 3 and 6.0% as grade 4. There were no deaths. When focusing on the worst grade of AEs experienced by each of the 32 patients, no significant association was identified between the worst grade of AEs and causative medication from the chi-square test (*p* = 0.661).

Of all 117 AEs, there were only 22 SAEs reported over 11 patients. However, three patients accounted for 13 (59.0%) of all SAEs. Patients hospitalized due to immunotherapy alone accounted for 50.0% of all SAEs, while 36.4% of SAEs were attributed to psychotropics, 4.5% to both immunotherapy and psychotropics, and 9.1% to neither. Regarding the worst grade of AEs experienced by each of the 32 patients, there was no significant association between SAE status and possible causative medication (*p* = 0.757).

## Discussion

Our study has demonstrated a significant reduction in CGI-S scores in patients with atypical and/or treatment-resistant psychiatric, regardless of whether they received immunotherapy (mean reduction = 1.2, 95% CI = 0.5–1.8, see Figure 1). Overall, a quarter of patients (9/36) showed a reduction in CGI-S score of three or more, indicating meaningful functional improvement (Figure 1(a)). There was no significant evidence that the size of this change depended on immunotherapy status, but this may be due to the small sample size of our study.

One concern, with the use of immunosuppression, is adverse effects including increased risk of infection. This study revealed no significant difference in the frequency of AEs or hospitalization with SAEs associated with immunotherapy compared to psychotropics in our cohort of 42 patients. Four patients accounted for 40% of the total AEs, each of whom received four or more immunotherapy agents and psychotropic classes, indicating their disease severity.

Immunosuppression, particularly the use of glucocorticoids, has significant AEs; however, current psychotropic medications also have significant metabolic side effects. In addition, there is a view that steroid-sparing immunosuppression has a high incidence of AEs. However, our study shows similar numbers of AEs with psychotropic medications and immunotherapy. The majority of our patients (61.9%) trialled three or more psychotropic classes, and 42.8% trialled three or more immunotherapy agents, highlighting the severity of their condition. In our study, the median time from initial psychiatric diagnosis to seeing an immunologist was 3.5 years (interquartile range [IQR] = 1.0–7.8 years), highlighting the need for better recognition of antibody-negative AIE in psychiatry. Hence, while serum investigations, brain imaging and EEGs may not always reveal observable abnormalities (Table 1), clinical suspicion followed by prompt and appropriate referrals to an immunology service to consider therapeutic trial, may enhance patient outcomes.

Since our study is underpowered, interpretation of our results is limited and requires replication in a larger study. In addition, a retrospective review of eMR has inherent limitations that compromise reliability due to potential errors in tracking patients’ outcome over time, interpreting clinical notes, missing data points, difficulty controlling confounding variables and risk of recall bias. The CGI-S scores are also constrained by their subjectivity, dependency on clinical judgement, inter-rater variability, limited sensitivity to subtle changes, lack of specificity for symptom domains and the categorical nature and simplicity of the scores.

Overall, we found that immunosuppression in addition to psychotropic treatment does not unduly increase risks of AEs and may have therapeutic benefit. This study highlights the need for clinical trials to study the safety and efficacy of immunosuppression in psychiatric patients, and we hope this contributes to better defining the risks and role of immunomodulation in psychiatry.
